# Nonoperative treatment of five common shoulder injuries: A critical analysis

**DOI:** 10.1007/s11678-018-0449-1

**Published:** 2018-02-19

**Authors:** Jonas Pogorzelski, Erik M. Fritz, Jonathan A. Godin, Andreas B. Imhoff, Peter J. Millett

**Affiliations:** 1 Steadman Philippon Research Institute, 181 West Meadow Drive suite 1000, 81657 Vail, CO USA; 2 Department of Orthopedic Sports Medicine, Technical University of Munich, Klinikum rechts der Isar, Ismaninger Str. 22, 81675 Munich, Germany; 3 The Steadman Clinic, 181 West Meadow Drive suite 400, 81657 Vail, CO USA

**Keywords:** Rotator cuff tears, Shoulder injuries, Tendinitis, Acromioclavicular joint, Humeral fractures, proximal, Rotatorenmanschettenläsionen, Schulterverletzungen, Tendinitis, Akromioklavikulargelenk, Proximale Humerusfrakturen

## Abstract

Economic pressure highlights the critical need for appropriate diagnosis and treatment of various shoulder pathologies since under-diagnosis and under-treatment can result in increased costs to society in the form of disability and lost production. On the other hand, aggressive over-treatment can further inflate already burgeoning health-care costs and potentially harm the patient. Therefore, it is crucial to distinguish the indications between operative and nonoperative management, especially in common shoulder pathologies such as rotator cuff tears, anterior shoulder instability, biceps tendinitis, lesions to the acromioclavicular joint, and proximal humeral fractures. As a result, a detailed analysis of individual risk factors for potential failures should be performed and treatment should be based on individualized care with consideration given to each patient’s particular injury pattern, functional demands, and long-term goals.

## Zusammenfassung

Der zunehmende Kostendruck in der Medizin verstärkt die Notwendigkeit einer rasch zielführenden Diagnose und Therapie verschiedener pathologischer Veränderungen im Bereich der Schulter. Unterversorgte Patienten erhöhen die Kosten für die Gemeinschaft durch längere Ausfallzeiten und damit erniedrigte Produktion, während überzogene Therapien die bereits ausufernden Kosten in der medizinischen Versorgung weiter erhöhen und den Patienten sogar potenziell schädigen können. Deshalb ist es unabdingbar, die Indikationen für operative und konservative Therapien zu kennen und anzuwenden, besonders im Hinblick auf häufige pathologische Veränderungen wie Rotatorenmanschettenläsionen, vordere Schulterinstabilität, Bizepssehnentendinitis, Akromioklavikular Gelenkluxationen und proximale Humerusfrakturen. Grundsätzlich ist es dabei wichtig, individuelle Risikofaktoren für ein Therapieversagen zu erkennen, den Erwartungshorizont des Patienten bezüglich funktionaler Ansprüche und Langzeitziele abzuklären und auch das Verletzungsmuster zu analysieren, um so letztendlich die Therapie individuell an den jeweiligen Patienten anpassen zu können.

## Introduction

Shoulder pain is one of the most common musculoskeletal complaints accounting for at least 4.5 million patient visits annually in the United States [43, 55] and occurring in as many as 51% of individuals in a lifetime [64]. Moreover, the economic burden of shoulder pathology is vast with annual direct costs for treatment of shoulder dysfunction totaling at least $7 billion in the United States, mostly due to operative treatment [47]. In Germany the percentage of affected patients and associated costs are expected to be similar. Moreover, with an aging and increasingly active patient population in the Western world, the absolute number of shoulder pathologies is likely to grow, further increasing costs.

These economic implications highlight the critical need for appropriate diagnosis and treatment of various shoulder pathologies, as under-diagnosis and under-treatment can result in increased costs to society with disability and lost production. On the other hand, aggressive over-treatment can further inflate already burgeoning health-care costs and potentially harm the patient.

Therefore, the purpose of this review is to distinguish the indications between operative and nonoperative management for five common shoulder pathologies, including rotator cuff tears, anterior shoulder instability, biceps tendinitis, lesions to the acromioclavicular (AC) joint, and proximal humeral fractures. Moreover, we aim to provide a short overview of the nonoperative management of each of these pathologies.

## Rotator cuff tears

### Indications for nonoperative treatment of symptomatic full-thickness rotator cuff tears

Although symptomatic rotator cuff tears are common and affect between 4% and 32% of the general population, the most appropriate therapy is still debatable [59, 75]. While there is agreement that traumatic rotator cuff tears should be treated operatively, the treatment choice for atraumatic rotator cuff tears remains unclear [38, 39]. This is mainly due to the fact that the radiological failure rate following rotator cuff repair surgery can be as high as 70% depending on the patient cohort, thus leading to the assumption that nonoperative treatment may be equivalent [5, 8, 24, 41]. This conjecture is further strengthened by the fact that pain relief and improvement of symptoms do not necessarily go hand in hand with structural healing of the tendon [59].

However, when taking a closer look at published outcomes in the literature, nonsurgical treatment appears to have limitations. While multiple studies with short-term follow-up of nonsurgical treatment show promising results with good clinical outcomes, studies with mid-term follow-up are more disillusioning [10, 22, 38, 39, 50]. This could be explained by the fact that smaller tears may not affect the force couples in the shoulder, thus a reasonable degree of shoulder function may be maintained [42]. As there is strong evidence that the natural history of nonoperatively treated rotator cuff tears leads to tear progression over time, nonoperative outcomes studies with longer follow-up may include more patients whose tears have progressed to the point of destroyed force couples [80].

Kukkonen et al. [38, 39] published a randomized controlled trial for the treatment of supraspinatus tendon tears in patients older than 55 years. A total of 180 shoulders with supraspinatus tendon tears were randomly allocated into one of three treatment groups:

1. Isolated physiotherapy
2. Acromioplasty and physiotherapy
3. Rotator cuff repair with acromioplasty and physiotherapy

After 1 year of follow-up, no statistically significant differences in outcomes were detected, thus leading to the conclusion that surgical therapy is not superior in these patients [38]. Later, with an additional year of follow-up, the groups still did not differ significantly in outcomes; however, tear progression measured with magnetic resonance imaging (MRI) suggested that only patients with lower physical demands should be treated nonoperatively and patient counseling is critical [39].

In another randomized controlled trial of 103 patients, which compared rotator cuff repair with nonoperative physiotherapy for tears not exceeding 3 cm, Moosmayer et al. [50] found several additional factors that may influence the outcome. With a minimum follow-up of 5 years, the results for the group of patients who had immediate tendon repair were generally superior to those of patients who underwent physiotherapy as primary treatment and decided later to progress with surgery. Furthermore, treatment failed in almost 24% of the patients who received physiotherapy as primary therapy, and they underwent subsequent rotator cuff repair. In 37% of patients who did not undergo surgery, the tear size increased more than 5 mm over 5 years with associated inferior outcomes [50].

Similar results were reported by Safran et al. [68], who followed up 51 patients younger than 60 years with full-thickness rotator cuff tears in a longitudinal study. In this particularly young patient cohort, almost half of the tears increased after a mean follow-up of 29 months. Moreover, the authors found a significant association between the size of the rotator cuff tear and pain, which led to the conclusion that young patients in particular benefit from surgery [68].

### Treatment

While multiple rehabilitation protocols for the postoperative treatment following rotator cuff repair have been proposed, there are only a few published studies focusing on treatment protocols for primary nonoperative management of rotator cuff tears [37, 48, 59, 75]. In general, conservative treatment options include 3–6 months of activity modification, physical therapy such as strengthening and stretching of the muscles of the shoulder girdle, and injection or oral anti-inflammatory and pain-relieving medication [37, 48, 59].

A prospective multicenter study published in 2013 by the MOON shoulder group of 452 patients treated with a standardized physical therapy program for atraumatic full-thickness rotator cuff tears revealed a 75% satisfaction rate in patients after 2 years of follow-up. Physical therapy included daily postural and stretching exercising as well as strengthening of the rotator cuff three times a week. If needed, patients were seen by a physical therapist, especially for manual mobilization of the glenohumeral joint. Although less than a quarter of patients underwent surgery in the short-term follow-up period, the lack of imaging follow-up raises doubts about the long-term success.

In summary, careful patient selection is necessary when nonoperative treatment for full-thickness rotator cuff tears is chosen. The best possible outcomes are generally achieved in patients presenting with pain as the primary symptom, those having largely intact coronal and axial force couples, and patients who are willing to trade functional deficits of their shoulder to avoid surgical risks. However, as there is no evidence that the torn tendon actually heals without surgical re-fixation, patient counseling about tear size progression is indicated. This includes the progression from an initially reparable tear to an irreparable tear, as well as inferior postoperative outcomes of chronic tears compared with acutely fixed tears. If treated nonoperatively, a combination of activity modification, stretching and strengthening of the periscapular muscles and the deltoid should be performed. MRI of a known rotator cuff tear can be performed on patients who want to progress with surgical refixation of the tear and those who wish to monitor tear progression to consider surgery at some future time point.

## Anterior shoulder instability

### Indications for nonoperative treatment of anterior shoulder instability

There is consensus in the literature that a detailed analysis of individual risk factors for recurrent instability should be made for each patient presenting with anterior instability to determine the most appropriate treatment [3, 61]. In general, known factors associated with a high risk of recurrent instability when treated nonoperatively are young age, an active lifestyle, bone loss of more than 20% of the glenoid surface, and engaging or off-track Hill–Sachs lesions[3, 9, 11, 44, 61, 65, 73].

In patients younger than 30 years of age, the risk of re-dislocation when treated nonoperatively is between 70 and 90% compared with up to 25% when treated operatively [9, 30, 71].

When nonoperative treatment is applied to overhead athletes and active patients, the re-dislocation rate is even higher [3, 61]. However, with increasing age, the re-dislocation rate in patients treated nonoperatively decreases substantially making nonoperative treatment an option [12].

**Fig. 1:**
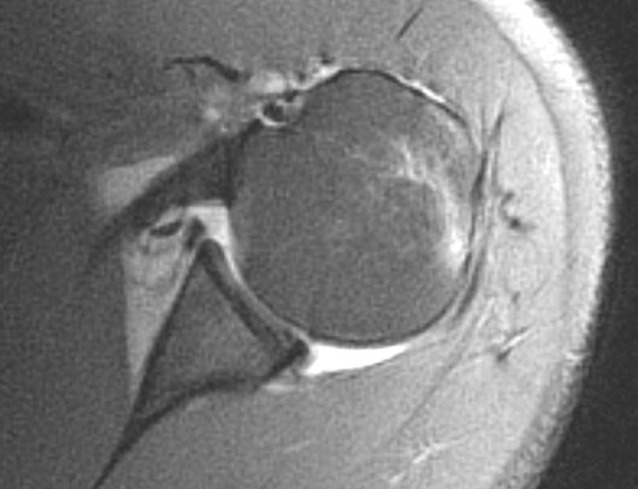
Axial T2-weighted magnetic resonance imaging sequence of a 36-year-old patient after a first-time shoulder dislocation. Given his age and the absence of any rotator cuff tear or other concomitant pathology, he was deemed low risk for re-dislocation. Therefore, nonoperative treatment was pursued, which was successful with no recurrent subluxation or dislocation

In general, patients without structural lesions of the glenohumeral joint can be treated nonoperatively, especially when older than 35 years (Fig. 1). However, the treating physician must ensure that concomitant injuries such as rotator cuff tears, Hill–Sachs lesions of more than 25% of the humeral surface, or glenoid bone loss are excluded as those would need surgical intervention [3, 11, 44, 66]. The “critical” amount of glenoid bone loss is typically defined as a loss of more than 20% of the glenoid surface [11, 44]. Another risk factor for recurrent instability is engaging or off-track Hill–Sachs lesions, as reported in recent literature recommending operative treatment [57, 73].

Furthermore, the injury pattern should be taken into account. High-energy trauma often results in a locked dislocation or displaced fracture of the glenoid or the humeral head and is generally best approached with surgical treatment. Finally, patients who have the ability to voluntarily dislocate their shoulder without discomfort should be treated nonsurgically in most cases, as these patients likely suffer not from structural instability but rather from functional instability, which can be due to a pathological functional activation pattern [27, 33] and may respond better to functional conservative treatments [70] or even electrical muscle stimulation in some therapy-resistant cases [51].

### Treatment

In order to manage shoulder instability without surgical intervention, a combination of immobilization and physical therapy is often used before the patient can return to activity [12, 35, 36, 54]. Physical therapy protocols may either follow a period of immobilization of about 3 weeks in internal or external rotation of the shoulder or be initiated immediately. The overall goal of physical therapy is to progress through glenohumeral strengthening and stabilization, thus reducing the probability of recurrent instability. Return to full activity is mostly allowed when there is symmetrical shoulder strength of the scapulothoracic and glenohumeral joints, as well as functional shoulder range of motion [12, 57].

More recently, several studies have focused on the position of the arm during immobilization after a traumatic anterior shoulder dislocation. In an MRI study by Itoi et al. [31], immobilization with the arm in external rotation resulted in reduction of the Bankart lesion after traumatic shoulder dislocation, thus supporting the hypothesis that immobilization in external rotation may be superior to immobilization in internal rotation. However, published clinical trials have not been able to demonstrate similar efficacy of external rotation immobilization for preventing recurrent shoulder instability [20, 78], including a recent randomized controlled multicenter trial published in 2014 [78]. Additionally, the conclusion that “immobilization in internal or external rotation does not change recurrence rates after traumatic anterior shoulder dislocation” was confirmed in a 2014 systematic review of the literature [76] and a 2016 meta-analysis of randomized controlled trials [77]. Of note, immobilization in external rotation is reported to be very uncomfortable and, therefore, could reduce patient compliance.

Overall, careful consideration of the injury mechanism, patient demands, and concomitant injuries associated with anterior shoulder instability are crucial when deciding on nonoperative vs. operative intervention. Patients younger than 35 years of age should rarely be treated nonoperatively as the recurrence rate is unacceptably high. If treated nonoperatively, immobilization in internal rotation seems to be more comfortable and shows equal outcomes to immobilization in external rotation and thus should be preferred, according to current literature findings.

## Biceps tendinitis

### Indications for nonoperative treatment of long head biceps tendinitis

Inflammation of the long head biceps tendon (LHBT) can lead to damage and weakening of surrounding supporting structures, thereby causing LHBT instability. In turn, instability can place increased stresses on the LHBT, which subsequently increase inflammation. This cycle can predispose the LHBT to rupture.

Given the potential success of nonoperative management for most LHBT tendinopathies, a management strategy involving medications and physical therapy should be the first step in treating these conditions. After progressing a patient through physical therapy, a course of nonsteroidal anti-inflammatory drugs (NSAIDs) and/or injections, it is important to re-evaluate the patient for progression of pain, weakness, and mechanical symptoms. At that time, continuation of a home exercise program vs. consideration of additional interventions will be discussed based on symptom progression.

**Fig. 2:**
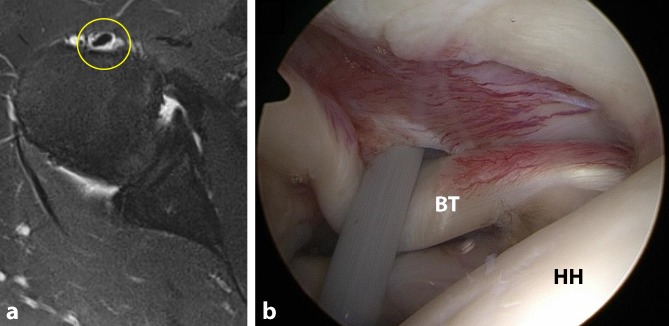
Images of a 46-year-old man with right-sided biceps tendonitis, diagnosed via history, physical examination, and **a** T2-weighted magnetic resonance imaging with a clear halo sign (*yellow circle*) around the long head of the biceps tendon indicating inflammation. The patient was treated conservatively with physical therapy and NSAIDs but continued to experience symptoms 6 months later. He thus underwent operative management as seen in **b** with the long head of the biceps tendon (*BT*) and biceps reflection pulley visualized through the standard posterior viewing portal. *HH* humeral head

If a patient progresses through all nonoperative treatment options and notes no improvement of pain or weakness, he or she should progress to surgical evaluation (Fig. 2). This is also the case for patients suffering from biceps reflection pulley lesions because these lesions do not heal and symptoms worsen over time. In general, patients suitable for surgical evaluation include the following: young, highly motivated patients with instability or complete LHBT rupture; manual laborers with significant instability or complete LHBT rupture; elite-level athletes with instability or complete LHBT rupture; any individual with a complete LHBT rupture who is not agreeable to a potential loss of elbow flexion or forearm supination strength and long-standing “Popeye” deformity; and any individual who has progressed through all stages of nonoperative treatment and continues to have symptoms of pain and/or weakness that affects their quality of life.

### Treatment

After identification of the underlying pathologic condition of the LHBT, treatment generally begins with activity modification, NSAIDs, and/or corticosteroid injections [1, 53]. NSAIDs can provide short-term benefit for swelling and pain control. However, there is little evidence that they are efficacious in treating chronic tendon injuries [13].

Use of corticosteroid injections should follow a similar treatment protocol to NSAIDs. Multiple case reports discuss the risk of tendon rupture with steroid injections, and caution should be exercised when injecting steroid around the LHBT [2, 13]. Corticosteroid injections alone will likely provide short-term anti-inflammatory effects for most LHBT disorders. However, they should be used for short-term pain relief and as an adjunct for the patient to initiate and tolerate a physical therapy program, rather than as a long-term treatment option. Because these injections have the potential to reach the glenohumeral joint, the anesthetic of choice, used in combination with corticosteroid, should be ropivacaine, as it is found to be less chondrotoxic than bupivacaine [62].

The initiation of a 3–6-month physical therapy program allows for progressive increase in muscle strength while providing protection against further LHBT and associated structure injury during rehabilitation [1, 4, 19, 53, 67].

Other evolving nonoperative treatment options for LHBT disorders include prolotherapy (dextrose solution, sodium morrhuate), platelet-rich plasma (differing concentrations of platelets, white blood cells, red blood cells, and activated and inactivated platelets), and stem cells (circulating stem cells, adipose-derived, bone marrow aspirate, bone marrow aspirate concentrate, amniotic membrane-derived). The choice to utilize one of these treatment options varies from patient to patient and condition to condition, and current research is beginning to thoroughly evaluate these interventions and to standardize treatment protocols [21, 23, 45, 46, 49]. Indications for these injections include pain impairing athletic performance, connective tissue laxity impairing athletic performance, and pain impairing rest and quality of life [49]. Future research is needed to determine which LHBT disorders respond best to, and what patient populations are the most suitable candidates for, such procedures.

## Acromioclavicular joint injuries

### Indications for nonoperative treatment of acromioclavicular joint injury

Injury classification is the single most important factor in determining the most appropriate treatment of acromioclavicular (AC) joint injuries. In 1989, Rockwood and colleagues developed the classification system that is most widely used for AC joint injuries today [79]. Notably, this system, which is based on the work of Tossy et al. [74], recognizes the importance of the coracoclavicular (CC) ligaments in joint stability [79].

**Fig. 3:**
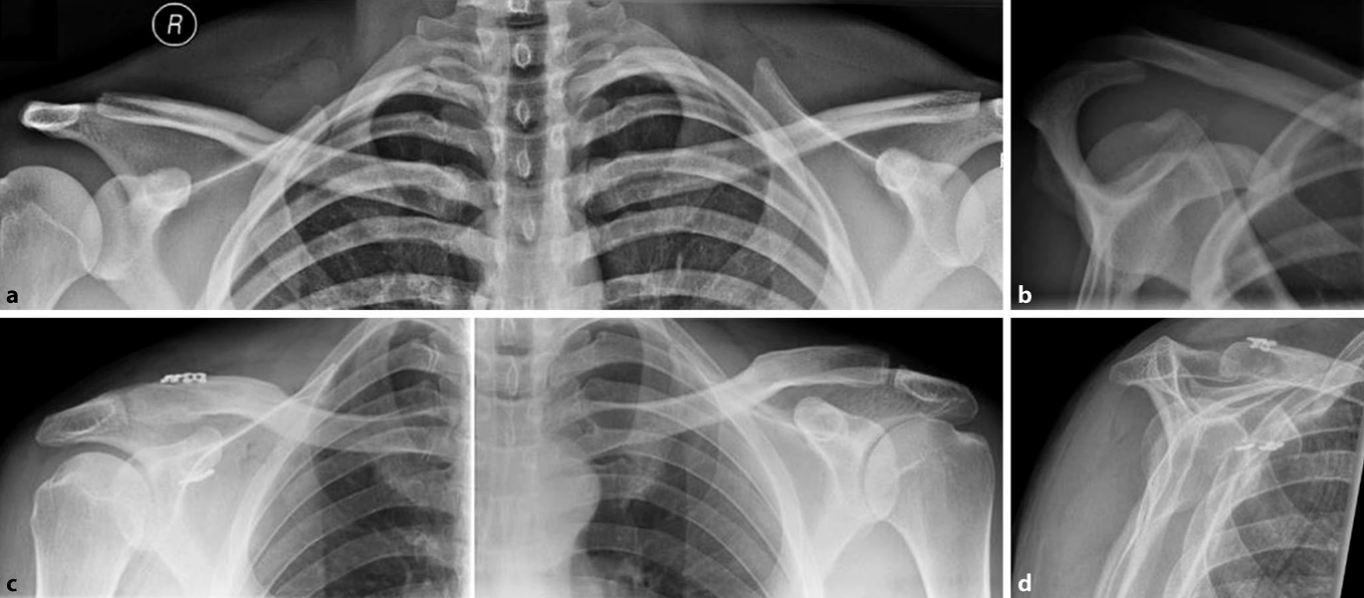
Radiographs of a 26-year-old male patient after a direct fall onto his right shoulder. **a** Panoramic view after injury showing a probable Rockwood type II injury. **b** However, the Alexander view demonstrates the clavicle overriding the acromion, thus indicating horizontal instability and defining this as a Rockwood type IV injury. Accordingly, the patient underwent operative therapy with two dog-bones instead of one in order to better address the horizontal instability, as pictured in **c**, the postoperative panoramic radiograph. **d** Postoperatively, the horizontal instability was resolved as demonstrated on the Alexander view 6 weeks after surgery

Rockwood type I injuries are characterized by a sprain without rupture of the AC ligaments with no anatomic dislocation and intact trapezius and deltoid fascia. Type II injuries involve rupture of the AC joint ligaments but are otherwise similar to type I. Type III injuries are characterized by rupture of both the AC and CC ligaments with superior displacement of the clavicle of 25–100% compared with the contralateral shoulder; notably, the trapezius and deltoid fascia are disrupted with this injury. Type IV injuries generally present with additional horizontal instability (Fig. 3). Type V injuries are similar to type-III injuries, but the clavicle is superiorly displaced more than 100% compared with the contralateral side. Type-VI injuries, which are rarely seen, involve rupture of both AC and CC ligaments with inferior displacement of the distal clavicle underneath the acromion; the trapezius and deltoid fascia are disrupted [74, 79].

Although high-level studies are rare in the orthopedic literature to definitively guide optimal treatment, there is a common consensus regarding the most appropriate treatments based on Rockwood type [6].

It is generally agreed that type I and II injuries should undergo initial nonoperative treatment while types IV–VI require surgery [6]. Optimal management of type III injuries has been controversial. In the highest-level study to date, the Canadian Orthopedic Trauma Society [16] recently completed a prospective randomized trial of 83 patients comparing nonoperative treatment of grade III, IV, or V AC joint injuries with operative intervention using a hook plate. Outcome scores at short-term follow-up as far as 2 years demonstrated no significant difference between the groups with the exception of superior radiographic results in the operative group [16].

Moreover, Petri and colleagues reviewed 41 patients with Rockwood grade III AC joint injuries who were initially treated nonoperatively [60]. Nonoperative management consisted of formal physical therapy two to three times per week for at least 6 weeks using a phasic approach with progression dictated by patient tolerance and evidence of improved scapulohumeral kinematics. Nonoperative treatment failed in 12 patients, who ultimately required surgery. Reasons cited for nonoperative failure included unremitting pain, weakness, instability, and dysfunction in spite of physical therapy. At a mean follow-up of 3.3 years, patient-reported outcome scores—including the American Shoulder and Elbow Surgeons score (ASES), Quick Disabilities of the Arm, Shoulder, and Hand score (QuickDASH), Single Assessment Numeric Evaluation score (SANE), and Short Form 12 Physical Component Summary (SF-12 PCS)—did not significantly differ between those who successfully completed nonoperative therapy and those who required eventual surgery [60].

In general, there is consensus that the horizontal stability of the clavicle is considered a potential key factor for a successful postoperative outcome. It is hypothesized that an unstable clavicle causes pain and functional deficits. Therefore, the ISAKOS shoulder committee [7] recently proposed a modification to the classic Rockwood classification in which type III injuries may be further subdivided into types IIIA and IIIB; type IIIA injuries are horizontally stable and may respond well to conservative management, but type IIIB injuries are unstable and should therefore be treated surgically [7].

### Treatment

Typical nonoperative treatment consists of primary immobilization and subsequent active rehabilitation [15]. However, evidence to support the efficacy of specific rehabilitation protocols is limited [15].

Gladstone et al. [25] published a physical therapy regimen for the nonoperative treatment of AC joint injuries types I, II, and III in athletes. Phase 1 lasts 3–10 days and focuses on elimination of pain and sling immobilization to protect the AC joint. Range-of-motion exercises begin in phase 2 with gradual progression of isotonic exercise for strengthening. Phase 3 involves advanced strengthening, and phase 4 involves sports-specific training before full return to activity [25]. The total length of rehabilitation can last 3–6 months. Moreover, it is important to check on the scapula movement since a significant number of patients suffering from AC joint injuries also present with scapula dyskinesis.

Overall, the general consensus regarding management of AC joint injuries is fairly straightforward: initial nonoperative treatment for Rockwood grades I–II, and operative intervention for grades IV–VI. For patients with grade III lesions, a closer look concerning the stability of the clavicle is necessary.

## Proximal humeral fracture

### Indications for nonoperative treatment of proximal humeral fractures

The number of bone parts and concomitant displacement mainly influences the treatment strategy of proximal humeral fractures. Nonoperative treatment of two-part fractures with early rehabilitation has been found to be at least as efficacious as surgical treatment in injuries with minimal displacement [29].

Better outcomes may be achieved with surgical fixation in cases with significant displacement, a bony avulsion of the supraspinatus tendon, a block to range of motion, and involvement of the anatomic neck. However, well-designed comparative studies of operative vs. nonoperative management of two-part fractures are lacking [26].

Some authors have found that greater tuberosity fractures with >5 mm of displacement may benefit from surgical fixation to reduce the risk of subacromial impingement [58, 63]. Lesser tuberosity fractures with internal rotation impingement may also benefit from surgery if nonoperative management fails [52]. In contrast to other parts of the proximal humerus, the anatomic neck is devoid of soft-tissue attachments and has a tenuous blood supply, which may result in an increased risk of osteonecrosis.

Court-Brown et al. recommend 2 weeks of sling immobilization followed by physical therapy for patients with two-part surgical neck fractures and valgus-impacted fractures [17, 18]. Two-part proximal humeral fractures with >66% translation were treated with either sling immobilization or with internal fixation with flexible intramedullary nailing and tension-band wires [17, 18]. No statistical difference was reported between the groups with regard to Neer score, return to activities of daily living, and union rates [17, 18]. The data demonstrate that the Constant score diminishes with advancing age and degree of displacement. However, when calculated based on age-adjusted Constant score, the older patients actually had better scores than the younger patients [14, 17, 18, 34]. Therefore, sling immobilization is an appropriate treatment option for patients older than age 60 years with valgus-impacted, two-part surgical neck or two-part tuberosity fractures.

Although three-part and four-part fractures often require surgical fixation, nonoperative management can be considered for patients with poor baseline function and/or an inability to tolerate surgery. In select three-part and four-part fractures, particularly valgus-impacted fractures with <1 cm of displacement of the tuberosities in relation to the head fragment, nonsurgical treatment may yield good-to-excellent results [17].

Although surgical treatment of complex fracture patterns is generally advocated, the efficacy of operative vs. nonoperative management remains to be clearly delineated. In a study of 60 elderly patients with a displaced three-part fracture of the proximal humerus, Olerud et al. found that surgical management with a locking plate resulted in better functional outcomes and health-related quality of life than did nonsurgical treatment, but at a cost of additional surgery in 30% of patients [56]. By contrast, a meta-analysis of randomized controlled trials did not find improved functional outcomes with open reduction and internal fixation (ORIF) compared with nonsurgical treatment in elderly patients with displaced three-part or four-part proximal humeral fractures [40]. The study concluded that these results must be considered in the context of variable patient demographics.

A systematic review supported the use of nonsurgical treatment of proximal humeral fractures and noted a 2% rate of osteonecrosis mainly associated with three-part and four-part fractures, high rates of radiographic union, and modest complication rates [32]. Ultimately, the patient’s baseline physiology and function may help to quantify the potential advantages of nonsurgical management, even in the setting of complex fracture patterns.

### Treatment

A number of proximal humeral fractures may be treated nonoperatively. However, patients must understand the expectations with this treatment approach and comply with the accompanying restrictions. In general, excellent results have been achieved with short-term immobilization (<2 weeks) in a sling and early physical therapy [28, 63, 72]. While the literature supports early mobilization, it is important to ensure that further fracture displacement does not occur. Sling immobilization with or without closed reduction also has a role in the management of displaced proximal humeral fractures [69].

## Practical conclusion

- For rotator cuff tears, the best possible outcomes with nonoperative therapy are generally achieved for patients presenting pain as the primary symptom of an atraumatic rotator cuff tear, largely intact coronal and axial force couples, and a willingness to trade functional deficits to avoid surgical risks.
- In patients suffering from anterior shoulder instability, careful consideration of the injury mechanism, patient demands, and concomitant injuries associated with anterior shoulder instability are crucial when deciding on nonoperative vs. operative intervention. Patients <35 years should rarely be treated nonoperatively.
- For tendinitis of the LHBT, treatment generally begins with a nonoperative treatment protocol including activity modification and NSAIDs. In patients with structural instability of the biceps tendon complex, or in any individual who continues to have symptoms of pain after nonoperative treatment, surgery is favored.
- The general consensus regarding management of AC joint injuries suggests initial nonoperative treatment for Rockwood types I–II, and operative intervention for types IV–VI. For patients with type III lesions, a pathologic instability of the clavicle potentially requiring surgical stabilization should be considered.
- Tuberosity fractures with >5 mm of displacement may benefit from surgical fixation to reduce the risk of subacromial impingement as well as displaced multifragment fractures in young and active patients.
